# Development and Piloting of a Scalable Training for Peer Recovery Specialists in an Evidence-Based Substance Use Intervention: Preliminary Implementation Outcomes

**DOI:** 10.1007/s10488-025-01464-9

**Published:** 2025-08-01

**Authors:** Morgan S. Anvari, Jessica F. Magidson, Salam Sulaiman, Eddie Killing, Dwayne Dean, Annette Dewbury, Logan Zelenak, Ellen Nixon, Andre Johnson, Julia W. Felton

**Affiliations:** 1 Department of Psychology, University of Maryland, College Park, MD USA; 2 Center for Substance Use, Addiction & Health Research, University of Maryland, College Park, MD USA; 3 Center for Health Policy & Health Services Research, Henry Ford Health System, Detroit, MI USA; 4 Detroit Recovery Project, Detroit, MI USA

**Keywords:** Peer recovery specialist, Behavioral activation, Training, Brief

## Abstract

Individuals from minoritized and under-resourced communities have significantly less access to specialized services from substance use disorder. Peer recovery specialists (PRSs) show promise for increasing access to services, especially in low-resource settings, but have not historically been trained to deliver evidence-based interventions (EBIs). While behavioral activation (BA) has shown promise as a PRS-delivered EBI, few studies have examined broader training efforts that may inform the scale-up of this model. This study describes the co-development (including PRSs and community-based treatment providers) and dissemination of a BA training for PRSs. The initial training was piloted with five PRSs, who provided qualitative feedback on training content and delivery. The revised training was then delivered to 168 PRSs. Post-training, participants completed implementation outcome measures assessing feasibility, acceptability, and appropriateness. A follow-up survey was sent within six months to assess continued use and perceptions of BA. Qualitative feedback identified BA as feasible for PRS delivery and appropriate for the PRS role, and identified ongoing supervision and experiential learning as key needs for PRS training. PRSs who received the revised training found it to be feasible, appropriate and acceptable. Follow-up surveys suggest PRSs continued to use BA skills and found it was a good fit to their role and feasible for their work situation. PRS-delivery of EBIs has the potential to increase access to treatment for individuals from low-resource communities. With appropriate modifications for the unique needs of this workforce, PRSs can be trained on a large-scale to deliver BA.

## Introduction

In 2022, only one in four people in need of substance use disorder (SUD) treatment in the United States received care (Substance Abuse and Mental Health Services Administration (SAMHSA), 2023). The gap between those who need and have access to treatment has disproportionately impacted historically underserved, ethnic/racially minoritized populations compared to White populations (Alegria et al., 2011; Larochelle et al., 2021). While numerous barriers have resulted in these inequities, foremost among them include SUD and SUD treatment, such as medication for opioid use disorder (MOUD), stigma (Anvari et al., 2022; Kleinman et al., 2023b), experiences of racial discrimination (Pro & Zaller, 2020), and discrepant access based on race (Stevens et al., 2022) and health insurance modality (Savitz et al., 2024). There is a pressing need to increase the number of behavioral health providers, particularly those who promote non-stigmatizing, compassionate approaches to SUD care. The current study describes the development of a training for peer recovery specialists (PRSs) in a person-centered, evidence-based intervention to reduce substance misuse.

### Peer Recovery Specialists

One promising approach to engage individuals in low-resource and minoritized communities is to engage the PRS workforce to increase access to services (Bassuk et al., 2016). PRSs are individuals with lived substance use and recovery experience, who are formally trained to support others in their efforts towards recovery. They are typically employed in various settings, such as hospitals/emergency departments, intensive outpatient programs, jails, and supportive housing (SAMHSA,  2009). PRSs also provide a variety of services, such as linkage to local resources, assistance in navigating the healthcare system, case management, and other non-structured general support (SAMHSA, 2009). Importantly, the PRS workforce has recently expanded dramatically, especially among community-based agencies that service low-resource areas.

Engagement with PRS services has been associated with various positive treatment outcomes, namely, increased treatment retention and satisfaction, improved relationships with treatment providers, and reduced substance use frequency and return to substance use (Eddie et al., 2019; Magidson et al., 2022). Qualitative work has demonstrated that patients often prefer working with someone with shared, lived experience over a provider who has “textbook knowledge” of substance use, and foresee increased rapport with a PRS than a traditional counselor (Anvari et al., 2022).

Given the promise of utilizing the PRS workforce to increase access to care and support positive substance use treatment outcomes in underserved communities, this workforce may be uniquely suited to deliver evidence-based interventions (EBIs). Yet, PRSs are not traditionally trained in disseminating evidence-based approaches that can flexibly incorporate their valued lived expertise. One EBI that has shown early promise for PRS delivery is behavioral activation (BA). BA is a manualized, brief EBI that was originally developed for the treatment of depression, though it has since been adapted for and demonstrated efficacy in the treatment of SUD (Daughters et al., 2018; Lejuez et al., 2011; Magidson et al., 2011, 2021). BA for SUD typically includes: (1) recognizing the negative cycle of thoughts, feelings, and behaviors, including urges and cravings; (2) learning to break this cycle; (3) identifying positively reinforcing, value-driven, substance-free activities that patients can feasibly engage in in place of substance use (Daughters et al., 2018). BA has specific promise for PRS delivery and low-resource settings due to its straightforward, flexible nature compared to other EBIs that require resource- and time-intensive training (e.g., cognitive behavioral therapy, dialectical behavior therapy, etc.). Our team has further adapted BA for SUD for PRS delivery, which includes integrating lived experience, community knowledge, and core elements of the PRS role, including recovery and motivational support into the delivery of core BA components. For further detail on qualitative work informing these adaptations, please see Kleinman et al., 2023, Magidson et al., 2020, & Satinsky et al., 2020. PRS-delivered BA has demonstrated feasibility, acceptability, and high PRS fidelity, as well as an association with improvement in SUD outcomes (Anvari et al., 2023a; Magidson et al., 2021, 2022).

### Training PRSs in Evidence-Based Approaches

Despite the emerging evidence supporting the utilization of PRS-delivered BA, there is little research to date regarding best practices for training PRSs in this EBI. Preliminary research has suggested that a brief, two-hour training may produce a significant increase in BA for SUD skills from pre-training to immediate post-training amongst PRSs (Anvari et al., 2023). However, other vital factors remain unknown, namely the feasibility of training PRSs in BA, as well as the perceived usefulness of the training, if/how PRSs implement these skills post-training, retainment of skills post-training, and how to train PRSs to deliver BA training. Further, research on training PRSs in motivational interviewing suggests that EBI trainings for PRSs are potentially effective when brief, but may require ongoing support and supervision to maintain long-term fidelity (Tsai et al., 2017).

### Current Study

The current study describes the development and preliminary implementation of a PRS-delivered BA training to increase widespread access to PRS-delivered BA, using a mixed-methods approach including qualitative and descriptive assessments of implementation outcomes guided by Proctor’s model of implementation outcomes (Proctor et al., 2011). Preliminary implementation outcomes assessed include appropriateness, feasibility, acceptability, and potential for sustainability (see Table 1). Within Proctor’s model, appropriateness, feasibility and acceptability are considered “early” implementation outcomes, whereas sustainability/maintenance is considered a “late” implementation model. Therefore, the current study assessed sustainability potential, in alignment with the goal of assessing preliminary implementation outcomes. The training was delivered by PRSs with personal experience delivering BA to individuals with SUD. Training recipients were PRSs either seeking certification or who were actively certified.

**Table 1:** Implementation outcomes

Implementation outcome	Description	Measurement
Appropriateness	PRS perception of fit with professional role	Focus Group Themes, Post-Training Survey
Feasibility	PRS-viewed compatibility with current tasks and activities	Focus Group Themes, Post-Training Survey
Acceptability	PRS satisfaction with elements of BA	Focus Group Themes, Post-Training Survey
Sustainability Potential	PRS perception of likelihood of maintaining BA implementation	Focus Group Themes, Post-Training Survey

## Methods

### Study Design

The team (including a certified PRS, licensed clinical psychologists, and a clinical psychology doctoral student) initially created a two-hour training based on preliminary data from a small PRS-led BA training pilot that evaluated change in BA skills in pre- and post-training via roleplays (Anvari et al., 2023). The training was first piloted with a group of PRSs (*n* = 5) who were recruited via word-of-mouth from a local outpatient SUD treatment setting and took part in a focus group following the training to guide iterative adaptation regarding format and delivery (more detail specified below). Feedback on training materials and procedures from the focus group were incorporated and subsequent changes were made to the training. The revised training procedures were then administered to the PRSs (*N* = 168) concentrated across two metropolitan areas– Detroit, MI and Baltimore, MD– both of which experience high prevalence of SUD (Maryland Department of Health, 2024; Michigan Department of Health and Human Services, 2024) and are historically under-resourced.

A total of 20 trainings conducted between Fall 2022 and Spring 2024 were co-led by either two certified PRSs (*n* = 14) or one certified PRS, who aided in the initial development of the training, and one clinical psychologist (*n* = 5), with the exception of the first training delivered to the focus group which a clinical psychologist conducted alone to guide early refinement. Each of the PRS trainers had in-depth experience with delivering BA through external experience not a part of this study and resided in Maryland (*n* = 1) or Michigan (*n* = 2). Each single-session training lasted approximately two hours. Content was derived from the manualized version of Brief Behavioral Activation Treatment for Depression (Lejuez et al., 2011) and BA for SUD (Daughters et al., 2016). Training content focused on six central intervention elements, described in Table 2. Additionally, trainers led discussions on how BA can be used as a manualized intervention or as a framework that various elements can be pulled from and integrated individually. Specific conversations around barriers to engaging clients in different BA elements were also included as a formal part of the training process.

**Table 2:** Intervention elements presented in training

Element	Description	Training techniques
Psychoeducation	Understanding the role of reinforcement in substance misuse	Content overview, role play, group discussion
Behavior Monitoring	Tracking daily activities and associated mood and substance use craving levels	Content overview, experiential exercise
Values and Activity Identification	Identifying personal life values and activities that align with those values	Content overview, role play, experiential exercise
Activity Scheduling	Scheduling valued activities into the day	Content overview, experiential exercise
SMART Goals	Setting realistic and achievable goals	Content overview, experiential exercise
Social Support Contracts	Identifying and clarifying with supportive people actions they can take to support client’s recovery	Content overview, group discussion

### Initial BA Training Development for PRS Delivery and Focus Group

Several considerations regarding training content and delivery were made to fit the unique needs of PRSs. The PRS role is unique in many aspects that may impact training needs. For instance, PRSs do not meet with clients in a traditional office-based setting as therapists do, rather, they often meet with clients in individualized settings to meet client needs (e.g., driving clients to appointments, in the emergency department, etc.). Given the flexible nature of the PRS role, it may be particularly important to train PRSs how to utilize BA in a flexible nature. Further underscoring the need for a PRS-tailored training, the PRS model is based in experiential learning and utilizing lived experience, rather than traditional clinical models of training and delivery. Organizations also vary greatly in their PRS supervision models (e.g., PRS-led, clinician-led) and frequency, as well as familiarity and accuracy with what falls within the scope of the PRS role (Felton et al., 2023), indicating a need to support PRSs who may have a less formal professional network. Finally, most EBIs do not formally include the sharing of interventionists’ lived experience. Given the centrality of this to the peer role, it is critical for any training to discuss how to share personal experiences within the larger context of a manualized intervention.

In alignment with the flexible nature of PRS work, this training presented BA as a set of tools that can be integrated into one’s typical work to further support their clients, as opposed to training PRSs in a manualized, session-by-session BA intervention. Core BA techniques including psychoeducation, behavior monitoring, values and activity identification, activity scheduling, SMART goals, and social support contracts (see Table 2 for greater detail) were taught both as stand-alone approaches and as elements within a larger, modular approach. Additionally, experiential learning approaches were utilized after the introduction of each element. For instance, trainees were asked to take a few minutes and write down everything they did that day and their corresponding mood and substance craving level (on a scale of 1 to 10). Trainers then had participants reflect on the exercise (how easy or hard it was, how informative it was, etc.) and whether they noticed any patterns in their own mood across activities. Trainers also facilitated a discussion regarding the formal incorporation of participants’ lived experience. Trainees were guided to identify personal experiences that would be appropriate to share at different points during the intervention. Finally, participants in training sessions were encouraged to get to know one another and share contact information (if coming from different organizations) to spur the creation of professional networks to support the ongoing use of BA. Opportunities to engage in discussion about BA content and implementation were woven in throughout.

#### Focus Group Participants

Focus group participants included five individuals who completed an in-person training and subsequent virtual focus group. Given the team’s previous qualitative work in the adaptation of BA for PRS delivery (Kleinman et al., 2023; Kleinman, Hines, Anvari et al., 2023a, 2023b, 2023c; Satinsky et al., 2020), this sample size was sufficient to further adapt training materials specifically. The only inclusion criteria were that participants were working as a certified PRS a Detroit-based substance use treatment center that is partnered with the research team, and operates a PRS training institute. Demographics of participants in the focus group are reported in Table 3.

**Table 3:** Demographic data for participants in the focus group and training group

	Focus group	Training group
Participant sex (female)	80%	65.6%
Parent age in years, *M* (SD)	44.2 (11.28)	47.68 (12.26)
Participant race/ethnicity		
White	80%	46.0%
Black	20%	42.5%
Multiracial	0%	7.5%
Other	0%	4.0%
Hispanic, Latine or of Spanish Origin	20%	4.0%
Current Employment		
Paid employment	100%	75.8%
Not employed, looking for work	0%	17.6%
Not employed, not looking for work	0%	3.6%
Not employed, looking for work in another occupation	0%	3.0%
Highest Grade Completed		
Less than high school	0%	0.6%
High school graduate/GED	40%	20.1%
Some college	0%	52.3%
College graduate	20%	17.2%
Master’s/Professional degree	0%	9.2%
Did not report	40%	0.6%

#### Focus Group Procedures

An announcement about the optional training and focus group opportunity was made at a staff meeting. Interested individuals were informed that participation in the training and subsequent focus group was voluntary and would not impact their employment. Focus group participants were initially administered the training and immediately afterwards took part in a 60-minute focus group. Focus group questions were developed by the researchers with input from PRSs and focused on asking participants about their preferred learning approaches (virtual vs. in-person trainings, inclusion of role plays, presentation of materials, and perceived needs for ongoing support for implementation), as well as their perception of the content and elements of BA. Further, focus group participants were asked to reflect on the importance of learning from another PRS versus a trainer from a different background or workforce. The focus group was led by a Ph.D.-level researcher and recorded and transcribed.

#### Qualitative Analyses

Using thematic analysis, the coding team (comprising of a doctoral student and a doctoral-level psychologist, both with extensive experience in qualitative analysis) utilized rapid qualitative analysis methods, modeled after Gale et al. (2019) who observed consistent findings between traditional, in-depth and rapid analysis. The initial coder (clinical psychology doctoral student) hand-coded the interview transcript identifying themes and sub-themes, and a second coder (doctoral-level psychologist) reviewed the coded transcript, making additional codes and changes to existing codes as needed. Thus, the interview was coded by one coder and reviewed by a different coder. Discrepancies were discussed and resolved via consensus.

### Revised Training Procedures

#### Revised Training PRS Participants

Following the focus group, the training was adapted to enhance delivery flexibility, experiential learning, supervisory/PRS networking needs, and further emphasize how to integrate the PRS role with BA delivery. Following this process, 168 PRS participants were enrolled in the two-hour training program. To improve accessibility, all trainings were offered through either a hybrid or fully virtual approach. As all trainings accommodated virtual attendance, PRSs were concurrently recruited from Maryland and Michigan. See below for greater detail on the training modifications made. The training was advertised through existing PRS networks within Maryland and Michigan, reflecting the geographic locations of the research team and PRS trainers. As such, recruitment efforts were limited to these regions, where the team had established community partnerships. While trainees were concentrated in metropolitan areas (Detroit and Baltimore), there were no formal inclusion criteria related to geography. Participants were primarily certified PRSs currently working for pay in the field (see Table 3 for a description of demographics and vocational experiences).

#### Post-Training and Follow-Up Surveys

All participants in the training sessions completed a demographics survey at baseline and an immediate post-training, self-report satisfaction measure (see below). All surveys were completed electronically via self-report. Participants were given continuing education credits approved by either Michigan or Maryland (depending on their state of employment) following completion of the post-training satisfaction measure for their completion of the training. Receipt of continuing education credits was identified as reasonable and valuable compensation for completing the training by the PRSs a part of the research team. Participants were later emailed a follow-up survey within 6 months after training to understand their ongoing use of the material. Participants received $5 in compensation for the brief follow-up survey. This training was deemed to be quality improvement by the Henry Ford Health Institutional Review Board and was therefore not required to obtain ethics approval.

#### Measures

##### Post-training Satisfaction and Implementation Potential

Participants completed a brief measure of training satisfaction created for the current study immediately post-training (see Supplementary Materials). Questions were developed to align with Proctor’s (2011) model of implementation outcomes. The 16-item measure utilized a 5-point Likert scale ranging from (1) strongly disagree to (5) strongly agree to assess the following subscales: (1) appropriateness for the PRS workforce (the extent to which participants agreed that the training content and delivery were appropriate for PRSs); (2) feasibility of implementing BA (perceived utility and practicability of training content to their work as PRSs; (3) acceptability of trainers (perceptions of the trainers’ effectiveness, teaching style, and responsiveness); and (4) potential for sustainability (perceived skill development and likelihood of using techniques in the future). Items within subscales were totaled and the mean level of individual items was also examined, with higher numbers indicating greater implementation potential (see Table 4 for items).

**Table 4:** Immediate and 3–6 month follow-up training assessment

Immediate post-training Measure	3–6 Month follow-up measure
1. Content was appropriate for Peer Recovery Coaches	1. I liked the BA training
2. Instruction was at a level appropriate to Peer Recovery Coaches	2. I was satisfied with the person that provided my BA training
3. Teaching methods were effective	3. BA seems like a good match (to the clients and situation I work in)
4. Visual aids, handouts, and oral presentations clarified content	4. BA seems doable (for the clients and situation I work in)
5. Knew the subject matter	5. I think evidence-based interventions, like BA, are important to use in my work.
6. Presented content effectively (e.g., promoted deep reasoning and learning; included a consideration of obstacles)	6. I have used at least one element of BA in my work (behavioral monitoring, activity scheduling, support contracts, etc.)
7. Presented content in an organized manner	7. I plan to start using at least one element of BA in my work in the future (behavioral monitoring, activity scheduling, support contracts, etc.)
8. Maintained my interest	8. I have applied at least one element of BA to my own life (behavioral monitoring, activity scheduling, support contracts, etc.)
9. Were responsive to questions, comments, and opinions	
10. Information could be applied to my work as a Peer Recovery Coaches	
11. My clients could benefit from my use of the information I learned today	
12. I foresee myself utilizing what I learned today when I work with clients	
13. I feel confident in using skills that I learned today in the future	
14. Teaching methods and tools focused on how to apply content to my work environment.	
15. How much did you learn as a result of this presentation?	
16. How useful was the content of this presentation for your professional development?	

##### Ongoing Utilization of BA

All participants were sent a post-training ongoing utilization measure within 6 months post-training. Responses were anonymous but participants were asked to inform us when they completed the measure and they received a $5 gift card to Amazon. This study-created survey included eight questions to assess participants’ ongoing utilization of BA (e.g., “I use at least one element of BA in my work”). All questions were measured with a 5-point Likert scale. Items were again aligned with implementation outcomes, including (1) satisfaction with training, (2) appropriateness for PRSs’ work; and (3) adoption of BA techniques for clients or self (see Table 4 for items).

#### Data Analysis

Descriptive statistics were evaluated to determine participant satisfaction and knowledge as well as ongoing usage. Given the aim of establishing preliminary implementation outcomes, inferential statistics were not utilized.

## Results

### Qualitative Findings

#### BA Components Are Feasible and Appropriate for the PRS Role and their Clientele

Participants described viewing BA as consistent with what they are already practicing with their clients, indicating both compatibility and fit with the scope of the PRS role. For instance, one participant described: “As far as identifying, like their triggers, connecting to the triggers and how they’re feeling that piece of it, I’ve done that with clients.” Another participant described that they informally utilize activity scheduling in their work with clients (without having previously identified this as activity scheduling).

Participants also described that they view BA as something that would be helpful for their clients. One participant also shared that they found the BA skills they learned to not only be potentially helpful for clients, but “very useful and powerful” in their own recovery as well. One participant described the value of behavior monitoring:

For me, it was like, the tracking part of it, that I’m thinking about what was shared, like, oh, you know, I like to clean in the morning. And when I’m cleaning, I like to listen to the news. But by the end of it, after listening to the news, you know, just kind of feel bad. I think that’s very important, because then it for me, and for others, like to be able to write down what it isn’t doing during the day, it might not be just get rid of everything. It just might be something that needs to be tweaked. I think that’s very important information like, oh, you know, talking to this person just doesn’t make me feel good. So tweaking that and maybe not talking to that person, or picking a different time of day to talk to that person. Like, it all makes a huge difference. I think like the scheduling and the tracking of that is very significant.

Lastly, one participant reported that BA components, such as homework, can aide with session planning and continuity:

Activities and sheets and daily tracking give something for to talk about in the session. And then it kind of helps conversation moving forward, you know, with like, the next sessions.

#### Perceptions of Training Format and Techniques

Overall, participants described satisfaction with training structure and procedures. While participants appreciated that the training was led by a PRS, there were mixed opinions on whether or not it is necessary for the training to be led by a PRS to be effective. In regards to training modality, one participant described that, given new virtual norms, having the option of joining the training via Zoom increases the accessibility of the training, so that “anybody and everybody, no matter where they’re at, can attend it.”

In regards to training content, participants reported that it would be helpful for future trainings to include roleplays and experiential components, with one participant stating:

So I personally, roleplay does give me like, a lot of anxiety. But as far as like memory goes, and my muscle memory, there’s something about me getting a brief little roleplay exercise in it gives me the confidence to know that I can do it, even though it’s not a real situation.

#### Need for Ongoing Supervision and Training Material Access for Sustainability

Participants offered various insightful suggestions to enhance future training sustainability and dissemination. To enhance sustainability, participants emphasized the importance of long-term supervision in the use of BA, which may include the need for supervisor-specific BA trainings.

Participants also noted that a key barrier to BA use was the inability to access worksheets and online materials. Participants suggested the creation of a website for PRS-delivered BA materials, and stated this might be helpful to enhance client uptake of BA as well:

So I have this image of the website, you know, that we were talking about earlier. You know, for the clients that I serve and stuff, it’d be great for them to be able to, like, go through a little bit of courses, you know, watch, like the testimonials, watch the videos, or, and, you know, like the trainings that we do, you know, maybe have some tests in there to really get the stuff soaked in and think about, you don’t have to print off like something for everybody to be able to go to, to get on board with it. You know, so it’s not just like, I go out to a training and I learned about it.

#### Incentivizing Training Attendance

Lastly, participants described incentives, such as testimonials and food items, may enhance motivation to enroll in a BA training. One participant also described that the BA training stood out to them against other continuing education courses due to being initially developed for depression:

And again, if I am remembering correctly, you all lead with this was actually a concept that has been used to treat or like combat the symptoms of depression. That stands out to you have my attention already. We’re fighting depression? Oh, great. Let’s use it. So just being very forward with the with the benefits of it and not having to work too hard because, you know, it seems like we can all agree how wonderful behavioral activation can be.

### Training Modifications

Following the qualitative feedback from focus group members, the research team made several adaptations to the training concerning both format and content. First, the team attempted to offer more virtual and hybrid training modalities to meet the varied needs of PRSs. Additionally, trainings were always led by at least one PRSs who could discuss their own experiences with providing BA to recoverees. Experiential exercises and opportunities for roleplays were woven into the content covering each BA element (see Table 2). Prompts were also included in the training curriculum for the PRS trainers to explicitly talk about how they used the intervention and how it worked for their clients (in line with the peer model of sharing lived experiences).

Additionally, a discussion of how to seek out supervision in delivering BA, including leveraging informal peer networks, was added to the end of the training to support PRSs in obtaining ongoing support. Trainers sought to facilitate connections among trainees using breakout rooms and interactive techniques (such as role plays) to spur connections as a way to sustain PRSs continued use of BA techniques. Trainers were also encouraged to support trainees in making connections to share knowledge of substance-free activities in their communities.

### Quantitative Findings

#### Post-Training Satisfaction and Implementation Potential

Of the 168 individuals who participated in the two-hour training, 131 completed the satisfaction and implementation potential measure immediately following training. Overall, participants reported finding the training appropriate, acceptable and feasible for PRS implementation immediately following the training. On a scale of 1 to 5, with 5 representing “strongly agree,” average ratings on items ranged from 4.75 (“visual aids and handouts clarified content,” *SD* = 0.72, range 1–5) to 4.84 (“how useful was the content of this presentation for your professional development,” *SD* = 0.42, range 2–5). Respondents were most enthusiastic about the training’s application to PRSs’ professional development and reported that they felt the techniques would generally apply to their work and benefit the individuals’ they worked with.

These results were in line with findings from the follow-up survey, completed 3–6 months following training. While all trainees were offered the follow-up survey, only 59 completed the measure. On average, respondents indicated that they were satisfied with the BA training and found it appropriate for their work. Importantly, 90% reported that “agreed” or “strongly agreed” that they had used at least one element of BA in their work and 86% reported that they had applied at least one BA element to their own lives.

## Discussion

The current study described the development and implementation of a training for PRSs on delivering an EBI, BA. While PRSs are uniquely suited to address disparities in access to SUD care for minoritized individuals in low-resource settings and are widely employed across many sectors and treatment settings, they have not typically received training in evidence-based methods that incorporate their lived experience. Training and supporting PRSs in deploying evidence-based approaches has the potential to help expand access to effective SUD treatment approaches and support the development of this workforce. Findings from this research suggest the suitability of an adapted BA training and the utility of this approach in supporting PRS-delivered EBIs.

Initial training procedures were created and developed by the current team, including researchers and practicing PRSs. The current team has completed substantial work in adapting BA for PRS delivery in resource-limited settings, including qualitative work to inform intervention adaptation (Kleinman et al., 2023; Kleinman, Hines, Anvari et al., 2023a, b, c; Satinsky et al., 2020), a feasibility-acceptability intervention pilot (Felton et al., 2023), a type 1 hybrid implementation-effectiveness pilot (Magidson et al., 2022), and ongoing adaptation and testing of the intervention across urban and rural communities. Qualitative findings supported the initial feasibility and acceptability of this training approach, with focus group participants reporting high perceived utility of BA, alignment with current work, and appreciation of training content and procedures such as experiential learning. Additionally, qualitative themes highlight some of the unique needs of this workforce, including opportunities for peer support to sustain BA implementation and utilizing experiential approaches to learning. This training framed BA as *either* a framework to be pulled from or a structured intervention to help PRSs in their work with clients, allowing for a more flexible view of EBI delivery that allows for incorporation of lived experience and unique PRS knowledge. Increasing the use of EBI components, even if not through a series of structured intervention sessions, has the potential to subsequently increase access to evidence-based care in underserved communities.

Results from the revised training and pilot of the training with 168 PRSs across two states suggest that participants found the format and content to be highly acceptable and appropriate for PRSs. Feedback gathered immediately following the training showed that this approach was congruent with the PRS role and applicable to their work. This finding is important because PRSs have reported their perceived importance of staying within the “peer lane”, which is recovery-oriented and meets patients where they are at in their recovery journey (Felton et al., 2023). Given BA’s flexibility, it lends itself well to be adapted to meet the needs of varying levels of recovery (Anvari et al., 2023).

Additionally, at the time of training, PRSs reported that they perceived this approach to be feasible and that they were likely to utilize BA principles with future clients. Importantly, data from our follow-up survey also suggests the possible long-term utilization of skills learned in this training. Though results are preliminary and examine a “low bar” to adaptation (the use of any BA elements), these findings provide early support for the potential sustainability of this training approach, especially if paired with ongoing supervision and peer support for implementation as participants suggested. Whereas more intensive training models of EBIs typically show promise for changing interventionist behavior (Frank et al., 2020), this study found preliminary evidence that a single BA training may lead to ongoing intervention adoption. If replicated, this is particularly important for supporting sustainability in underserved, low-resource settings.

### Clinical Implications

It is vital to implement innovative strategies to narrow the gap between those who need and have access to treatment, particularly within underserved, minoritized populations that are disproportionately impacted by the treatment gap (Alegria et al., 2011; Larochelle et al., 2021). Beyond mere access to treatment, it is also crucial that treatment approaches are culturally acceptable and feasible within the communities receiving them. Indeed, the PRS workforce may be one particularly promising solution for expanding the substance use workforce (Bassuk et al., 2016) and bolstering acceptability through their shared, lived experience (Anvari et al., 2022; Kleinman et al., 2023a; Satinsky et al., 2020). Thus, expanding access to PRS training in flexible, straightforward EBIs such as BA show particular promise in expanding access to EBIs for substance use and supporting substance use treatment outcomes. These preliminary training outcomes provide an important stepping stone for the widespread dissemination of a PRS-delivered BA training, and, subsequently, expanding access to EBIs for substance use within low-resource, underserved communities.

### Limitations and Future Directions

As this study reports on descriptive outcomes and qualitative findings from one focus group, generalizations cannot be made beyond the sample, and claims regarding causality cannot be made. Among the larger trainee sample, a smaller percentage of individuals completed the follow-up survey than the immediate post-training survey, which may impose a bias in the descriptive results. Additionally, all surveys were completed anonymously, so follow-up responses could not be compared or linked to the immediate post-training survey. Future research would also benefit from examining potential moderators of training outcomes, including PRS attributes (e.g. motivation to learn new techniques), PRS location (e.g. state credentialing rules, work environments), patient populations (e.g. the types of patient populations PRSs work with) and training characteristics (e.g. PRS vs. clinician-led trainings). Although PRSs reported their ongoing use of BA, this study did not include an objective measure of PRS fidelity to BA delivery. Future research should conduct a more in-depth assessment of ongoing BA usage, including examining PRS fidelity to BA post-training. Additionally, utilization of an objective measure of ongoing use following training, and examination of patient experiences and treatment gains, would further clarify the utility of this approach to ultimately address disparities in access to EBIs.

Based on focus group feedback, the creation of a supervisor manual and training is vital for the sustainability of PRS-delivered BA. Future work may explore sustainable supervision frameworks for PRS-delivered BA, such as a tiered model where senior PRSs provide routine supervision to other PRSs, supported by consultation from licensed clinical psychologists. Informed by focus group feedback emphasizing the importance of ongoing supervision and learning, supervision structures may integrate both individual and group formats to facilitate reflective practice, skill reinforcement, and sustained intervention fidelity. Open access to BA materials (e.g., training slides, homework sheets, etc.) is a necessary next step in increasing the accessibility of BA skills and training.

## Conclusions

PRS-delivery of EBIs has the potential to increase access to behavioral treatment for individuals who have historically not received evidence-based approaches. Results from this paper suggest that, with appropriate modifications to reflect the unique needs of this workforce, PRSs can be trained on a large-scale to deliver BA. These results hold promise for future scale up and subsequent applications for behavioral health conditions more broadly in addition to SUD, for example, BA for depression. It is important that future work continue to develop best practice guidelines for widescale PRS trainings for EBI delivery to expand access to adequate, culturally response care.
